# Development and validation of a Solution-Focused Group Therapy program for interpersonal relationships in adolescents with anxiety and depression: a Delphi-based consensus study

**DOI:** 10.3389/fpsyg.2026.1834496

**Published:** 2026-07-22

**Authors:** Yanan Gu, Cong Zhang, Sinuo Zhang, Xueyun Wang, Qian Zhuang, Hengqian Wu, Junjing Zhu, Fan Hai, Lixia Zhang

**Affiliations:** 1 The Eighth People’s Hospital of Zhengzhou, Zhengzhou, China; 2 Department of Education, Monash University, Clayton, VIC, Australia

**Keywords:** adolescents with anxiety and depression, Delphi method, expert consensus, interpersonal relationships, Solution-Focused Group Therapy (SFGT)

## Abstract

**Objectives:**

To develop and validate a standardized, de-pathologized Solution-Focused Group Therapy (SFGT) program specifically designed to address interpersonal dysfunctions among Chinese adolescents with anxiety and depression.

**Methods:**

A two-round Delphi method was employed to integrate multidisciplinary perspectives. An initial set of intervention components was developed based on the systematic literature review and semi-structured interviews with 10 frontline practitioners. This was followed by two iterative evaluation rounds involving a purposive panel of 20 experts in psychiatry, clinical psychology and education, all situated within the context of the high-pressure educational system in central China. Consensus was quantified using the expert positivity coefficient, authority coefficient (*Cr*) and Kendall’s coefficient of concordance (*W*), with refinements guided by the principles of clinical safety, cognitive adaptation and ethical boundaries.

**Results:**

Both consultation rounds achieved a 100% response rate, yielding a high expert authority coefficient (*Cr* = 0.90). The consensus trajectory showed improvement, with Kendall’s *W* increasing from 0.211 to 0.251 (*P* < 0.001) and all final-round coefficients of variation (*CV*) falling below the 0.25 threshold. Key qualitative modifications included the exclusion of competitive tasks to mitigate acute rejection sensitivity and the reframing of the Miracle Question into the Small Change Hypothesis to accommodate the diminished cognitive processing speed characteristic of depressive states. The final protocol comprises eight functional units structured to foster interpersonal resilience.

**Conclusion:**

The developed SFGT program demonstrates preliminary content validity and theoretical plausibility. The protocol is hypothesized to activate therapeutic factors such as universality and corrective emotional experiences, which may contribute to reducing learned helplessness and mitigating social stigma among adolescents in the Chinese cultural context. However, empirical validation of these hypothesized mechanisms—including clinical efficacy, dose–response relationships, and long-term interpersonal outcomes—requires rigorous testing through multi-center randomized controlled trials and implementation studies.

## Introduction

Adolescent mental health has currently reached a global tipping point, with the rising incidence of anxiety and depression posing a formidable challenge to public health systems worldwide. Amid this crisis, interpersonal impairment emerges not merely as a symptomatic byproduct of emotional distress, but as a pathogenetic engine that sustains and exacerbates internalizing disorders (Loades et al., 2020). Whether manifested through peer exclusion, social withdrawal, or pervasive familial conflict, these relational disruptions trap adolescents into a cycle of emotional vulnerability that often resists standard pharmacological or brief interventions (Chen et al., 2005). Consequently, there is an urgent need to redirect therapeutic focus toward the interpersonal domain, which serves as both a primary stressor and a potential site for systemic recovery (Racine et al., 2021).

The landscape of adolescent distress in China is uniquely shaped by a socio-educational context defined by extreme academic competition and hierarchical family structures (Li et al., 2022). Within this environment, emotional fluctuations are frequently interpreted by parents through a strictly pathological lens (Bond et al., 2013) who often demand an immediate clinical cure to prevent academic derailment (Wang et al., 2025). Paradoxically, this intense evaluative pressure reinforces adolescent’s psychological defenses and amplifies social stigma, rendering traditional etiology-focused therapies—which delve deep into the origins of problems—potentially counterproductive (Steare et al., 2023). Such models may inadvertently intensify attributional conflicts between parents and children, contributing to high attrition rates in clinical settings as families struggle under the dual burden of academic demands and intensive psychotherapy (Luo et al., 2024).

Solution-Focused Brief Therapy (SFBT) emerges as a compelling alternative in this context, offering a postmodern, de-pathologized stance that aligns with the need for efficient intervention (Franklin et al., 2017). By shifting focus from problem analysis to resource activation, SFBT employs techniques such as exception-seeking and the Miracle Question to foster a sense of perceived control. For adolescents grappling with learned helplessness, this emphasis on micro-successes provides an immediate boost to self-efficacy without the stigmatizing weight of traditional diagnostic labels (Chen et al., 2023). Crucially, the not-knowing stance inherent in SFBT helps neutralize the adversarial power dynamics prevalent in Chinese families, transforming the therapeutic space into one of collaborative exploration rather than expert-led correction (Ramos-Heinrichs, 2023).

The effective implementation of SFBT with adolescents requires practitioners to possess appropriate professional qualifications and specialized training. In the Chinese mental health service context, three categories of professionals are considered qualified to deliver SFBT interventions to adolescents. First, nationally certified psychotherapists, a recognized health technical profession in China, may conduct SFBT interventions after completing systematic training, including didactic instruction in SFBT theory (International School Counselor Association, 2024) and a recommended minimum of 20 h of supervised practicum or equivalent continuing education credits completed within the past 5 years (National Health and Family Planning Commission, 2013). Second, psychiatrists or pediatricians with specialized SFBT training may also implement these interventions, drawing upon their diagnostic authority and medical expertise to manage comorbid conditions. Third, school psychological counselors holding a master’s degree or higher in psychology or education who have completed SFBT workshop training may serve as co-therapists or implement adapted versions of the protocol in school settings (Chen et al., 2018). Importantly, these professional groups have distinct clinical roles and boundaries: psychiatrists hold diagnostic authority; psychotherapists are qualified to conduct crisis intervention and manage acute psychological crises (e.g., self-harm, suicidal ideation, acute anxiety episodes); and school counselors—who lack diagnostic authority—must operate under the supervision of psychiatrists or psychotherapists and follow a clear emergency referral protocol when acute crises arise. This tiered qualification framework ensures both the quality of intervention delivery and the safety of adolescent participants across diverse service settings.

While brief psychological interventions have demonstrated efficacy in reducing anxiety and depressive symptoms among youth, with single-session and time-limited interventions showing promising effect sizes across diverse settings (Chen et al., 2023), existing solution-focused protocols for adolescents remain fragmented. Specifically, three research gaps persist: first, the lack of a standardized SFGT protocol tailored to the interpersonal needs of adolescents with anxiety and depression; second, insufficient cultural adaptation of SFBT techniques to the Chinese family context, where high academic pressure and collectivist values shape interpersonal dynamics; and third, the absence of expert consensus on intervention structure, safety boundaries, and the adaptation of core techniques with diminished cognitive processing speed associated with depressive states.

The present study seeks to bridge this gap by conducting a two-round Delphi expert consultation (Jafri et al., 2022; Jünger et al., 2017). The innovation of this study lies in three aspects: (1) the systematic integration of rejection sensitivity considerations into activity design, replacing competitive tasks with collaborative alternatives to prevent therapeutic re-traumatization; (2) the adaptation of the Miracle Question into the Small Change Hypothesis, which reduces cognitive demands and enhances perceived controllability for adolescents experiencing learned helplessness; and (3) the establishment of a standardized eight-unit framework that bridges group therapeutic factors with real-world interpersonal skill transfer. Our goal is to provide a scientifically validated tool that not only addresses the immediate interpersonal needs of adolescents with anxiety and depression but also strengthens the broader social support infrastructure essential for long-term mental resilience.

## Materials and methods

### Research design and ethical considerations

This study is a methodological study employing a two-round Delphi expert consultation for the development and content validation of a structured Solution-Focused Group Therapy intervention protocol. The Delphi technique was selected for its established utility in healthcare research for gathering expert perspectives and building consensus when definitive evidence is not yet available. The study procedures were guided by the CREDES (Guidance on Conducting and REporting DElphi Studies) recommendations (Jünger et al., 2017) and established Delphi methodological frameworks (Carlisle et al., 1997). The research was conducted in accordance with the Declaration of Helsinki and approved by the Medical Ethics Committee of Zhengzhou Eighth People’s Hospital (No. 2024-KY-005). Informed consent was obtained from all participating experts, and measures were implemented to ensure anonymity and data confidentiality throughout the process. All participating experts took part voluntarily without financial compensation.

The Delphi technique was selected for this study because it is specifically designed for expert consensus-building and content validation in the absence of definitive evidence, not for end-user acceptability testing. Consequently, adolescents themselves were not included in the expert panel. School counselors were also not represented in the panel, as the primary focus of this Delphi was clinical and therapeutic expertise; however, the protocol’s applicability to school settings remains an important avenue for future adaptation.

### Expert panel selection

This study employed purposive sampling to recruit a panel of experts between June and August 2024. The panel included professionals working in psychiatric hospitals, psychological departments within general hospitals, and university counseling centers in Henan Province, China.

#### Expert identification and invitation

Potential experts were identified through two primary pathways: (a) the research team’s attendance at SFBT academic conferences within Henan Province, where relevant scholars were noted; and (b) the team’s engagement in provincial academic exchanges and training activities, through which experts with experience delivering SFBT groups or related group interventions in school or hospital settings were identified. Using purposive sampling combined with these identification strategies, a total of 24 potential experts meeting the inclusion criteria were initially identified and invited via email and telephone. Of these, two declined due to scheduling conflicts arising from out-of-town work commitments, one did not respond to the invitation, and one initially accepted but was unable to complete the questionnaire due to workload pressures. The final panel comprised 20 experts. Both Delphi rounds achieved a 100% questionnaire return rate (20/20), with no expert attrition.

#### Expert panel composition

Among the 20 final participants, 13 were from hospital-based clinical settings (psychiatry and psychotherapy) and seven were from university counseling centers with educational psychology backgrounds.

#### Expert panel size justification

The number of experts in Delphi studies has no universally agreed-upon standard in methodological literature. Commonly cited recommendations range from 15 to 50 experts (Diamond et al., 2014); other sources suggest that 5–20 experts with diverse expertise are sufficient (Rowe and Wright, 2001). A panel of 20 experts falls within the optimal range of these recommendations, balancing disciplinary representation with procedural feasibility.

Inclusion criteria were defined as follows: (1) A minimum of 10 years of professional experience in psychiatry, psychotherapy, or education; (2) Possession of a bachelor’s degree or higher. (3) Holding a senior professional title. To accommodate regional practicalities, psychological therapists with intermediate titles were considered eligible if they possessed over 10 years of specialized clinical experience. For the seven experts from the field of education, all held at least a bachelor’s degree in educational psychology or related discipline, with a minimum of 10 years of experience in school-based mental health services. Their qualifications were comparable to those of hospital-based experts in terms of degree attainment and length of clinical experience.

### Development of the initial item pool

The research team initiated the process by conducting a systematic search of domestic and international databases (including CNKI, Wanfang Data, PubMed, Web of Science, and Cochrane Library) using terms such as “Solution-Focused Brief Therapy,” “Adolescent Depression,” “Adolescent Anxiety,” and “Group Therapy.” A core group of four psychotherapists and two psychiatrists then drafted a preliminary framework, synthesizing insights from existing literature and localized clinical observations in China.

Subsequently, the group developed an interview outline designed to explore three key areas: (1) Identifying distressing scenarios and common defense mechanisms in adolescents’ peer and family relationships; (2) discussing the potential application of normalization and reframing techniques for individuals experiencing learned helplessness; (3) exploring appropriate timing for introducing conflict-resolution strategies.

Semi-structured interviews were subsequently conducted with 10 frontline clinicians (comprising four psychiatrists with over 5 years of clinical experience and six psychotherapists with a minimum of 3 years of individual and group psychotherapy experience, all working in adolescent mental health) to gather qualitative insights. The interviews were conducted by four mid-level psychotherapists from the research team, each with at least 5 years of clinical experience in child and adolescent psychiatry, who followed a standardized interview guide covering interpersonal distress scenarios, defense mechanisms, and applicability of SFBT techniques. The resulting initial draft comprised the program’s overall objectives, eight structured units (each consisting of warm-up, core activities, and closing segments), and suggested applications for seven core SFGT techniques.

### Data collection and evaluation criteria

Questionnaires were distributed in person to collect demographic information and assess experts’ familiarity with SFBT. Experts were asked to rate the perceived importance and feasibility of the program goals using a five-point Likert scale. They also evaluated the themes, coherence, and safety of the eight intervention units, as well as the adaptability and applicability of the core SFGT techniques.

A “suggestions and comments” section was also included to encourage detailed feedback on the proposed activities. Additionally, experts were surveyed regarding their familiarity with the evaluation indicators and the basis of their judgments. The full expert consultation forms for Round 1 and Round 2 are available in the Supplementary material.

### Anonymity and feedback protocol

To minimize selection bias and ensure the integrity of the Delphi process, experts were anonymized to one another throughout the study. Each expert was assigned a unique code known only to a dedicated research assistant, who distributed and collected all questionnaires without revealing individual identities to other panel members. No face-to-face meetings or group discussions were held among experts. The feedback summary provided after Round 1 presented aggregated statistical results (*M*, *CV*, and summarized qualitative themes) without any identifying information about which expert contributed which comment. No experts withdrew from the study; both rounds achieved a 100% response rate.

### Consensus criteria

Indicators were considered for modification or exclusion if they met any of the following criteria: a mean score (M) < 4.0 for importance, feasibility, or safety; a coefficient of variation (*CV*) > 0.25. Qualitative feedback highlight safety concerns or incompatibility with adolescent cognitive development. After each round, summarized statistical feedback were provided to expert panel to inform their subsequent evaluations.

#### Methodological rationale

Delphi versus content validity index (CVI). We acknowledge that the content validity index (CVI) is a widely used method for content validity assessment in instrument development (Polit and Beck, 2006). However, CVI is a static, one-time content validity evaluation method that typically requires experts to rate each item’s relevance to the measurement construct independently. Its core characteristics are: (a) independent item evaluation without considering inter-item relationships; (b) no iterative feedback mechanism; and (c) suitability for scales or questionnaires with clear, independent item structures.

In contrast, the Delphi method is a dynamic, iterative consensus-building approach characterized by: (a) multi-round anonymous feedback allowing experts to adjust their judgments after understanding the distribution of group opinions; (b) simultaneous evaluation of multiple interrelated dimensions (importance, feasibility, safety, appropriateness, operability); and (c) integration of quantitative ratings with qualitative feedback for iterative protocol optimization.

Our intervention protocol comprises eight interconnected therapeutic units with thematic progression (from trust-building → cooperative awareness → self-discovery → communication skills → conflict resolution→emotion regulation → social support → separation management). These units are not isolated “items” but exhibit content coherence and logical progression. Content validation of such a multi-dimensional, interrelated protocol requires experts to evaluate each component within the context of the overall framework and to converge toward consensus through iterative feedback. Critically, our unit of analysis is the protocol, rather than individual units within the protocol. *CVI* can indicate whether each unit is important independently, but it cannot capture higher-order decisions—such as whether a competitive activity should be entirely replaced—that emerge from experts’ holistic understanding of the protocol’s overall structure and internal logic. This is precisely the advantage of the Delphi method—it enables systematic convergence of expert opinion through multi-round anonymous feedback. The combination of mean scores, *CV*, and Kendall’s *W* allowed us to dynamically track the consensus convergence process (from *W* = 0.211 to 0.251), while integration of qualitative feedback enabled substantive protocol optimization based on experts’ specific suggestions.

### Statistical analysis

Data analysis was performed using SPSS version 22.0. Expert Engagement was assessed through questionnaire recovery rates and the frequency of qualitative suggestions.

#### Expert authority coefficient (*Cr*)

The expert authority coefficient was calculated as *Cr* = (*Ca* + *Cs*)/2, where *Ca* represents the judgment basis and *Cs* represents familiarity with the subject matter. *Ca* was derived from experts’ self-rated basis of judgment (theoretical analysis, practical experience, and literature review), and *Cs* was rated on a five-level scale from “unfamiliar” (0.2) to “very familiar” (1.0). A *Cr* ≥ 0.70 was considered indicative of acceptable reliability (Shi et al., 2022).

#### Consensus level

Inter-expert agreement was evaluated using Kendall’s Coefficient of Concordance (*W*), where values range from 0 (no agreement) to 1 (perfect agreement), with higher values indicating greater agreement among experts. Significance was tested using the χ^2^ test, with *P* < 0.05 indicating statistically significant concordance (Schmidt, 1997).

The reporting of the Delphi process adheres to the CREDES (Guidance on Conducting and REporting DElphi Studies) guidelines (Jünger et al., 2017).

### Finalization of the intervention program

Each intervention unit is designed to last approximately 90 min, comprising a 15-min warm-up phase to establish group cohesion and reduce initial anxiety, a 60-min core activity phase focused on the session’s thematic content, and a 15-min closing phase for reflection and goal setting. Based on expert consensus, the recommended group size is 6–8 adolescents per session, a range that allows for meaningful interpersonal interaction while remaining manageable for a single facilitator. The thematic content of each unit is summarized in Table 4.

## Results

### Expert panel characteristics

A total of 20 experts participated in the consultation. Their demographic and professional profiles are presented in Table 1. The mean age of panel was 47.05 ± 3.53 years, with an average of 22.5 ± 3.61 years of professional experience. Notably, 85% of the experts held a master’s degree or higher, and 75% obtained senior professional titles. In addition, 70% of the participants reported having a professional background in SFBT.

**TABLE 1:** Summary of expert panel demographics (*N* = 20).

Item	Category	Number (*n*)	Percentage (%)/(x̄ * s)*
Gender	Male	9	45
	Female	11	55
Mean age			47.05 ± 3.53
Mean working years			22.50 ± 3.61
Academic degree	Bachelor	3	15
	Master	13	65
	Doctorate	4	20
Professional title	Intermediate	5	25
	Associate senior	7	35
	Senior	8	40
Professional field	Psychiatry	8	40
	Psychology	5	25
	Education	7	35
Institution type SFBT background	Universities	4	20
	Psychiatric hospitals	9	45
	General hospitals	3	15
	Research institutes	4	20
	Yes	14	70
	No	6	30

### Reliability of expert consultation

Expert engagement was demonstrated by a 100% response rate across both rounds. In the first round, six experts provided qualitative suggestions, reflecting a high level of active involvement. The expert authority coefficient (Cr) was calculated at 0.90, derived from a mean familiarity (Cs) of 0.86 and a judgment basis (Ca) of 0.95. These values suggest that the expert evaluations provided a reliable foundation for the study.

### Coordination of expert opinions

Following two rounds of consultation, expert opinions demonstrated a clear trend toward convergence. In Round 1, mean importance scores for core goals ranged from 3.90 to 4.75 (*CV*: 0.09–0.29). After revisions based on expert feedback, Round 2 yield mean importance scores increase to 4.30–4.95, while *CV* values narrowing to 0.06–0.11. Notably, the *CV* for technical operability and feasibility significantly decreased, with all indicators in the final round falling below the predefined threshold of 0.25 (Table 2). Kendall’s *W* increased from 0.211 (χ^2^ = 198.783, *P* < 0.001) in Round 1 to 0.251(χ^2^ = 235.484, *P* < 0.001) in Round 2. While the change was statistically significant, the absolute increase was relatively small. According to conventional benchmarks (where *W* > 0.5 typically indicates strong consensus), the expert panel achieved a moderate level of agreement by the second round.

**TABLE 2:** Statistical comparison of evaluation dimensions between two rounds.

Evaluation object	Statistic	Round 1	Round 2
Core goals	Importance (*M/CV*)	3.90–4.75/0.09–0.29	4.30–4.95/0.06–0.11
	Feasibility (*M/CV*)	4.20–4.80/0.09–0.30	4.10–4.85/0.08–0.20
Program units	Importance (*M/CV*)	4.05–4.80/0.09–0.26	4.20–4.95/0.05–0.17
	Feasibility (*M/CV*)	3.25–4.70/0.10–0.33	4.10–4.90/0.06–0.18
	Safety (*M/CV*)	3.50–4.65/0.11–0.35	4.05–4.95/0.05–0.20
SFGT techniques	Appropriateness (*M/CV*)	3.20–4.65/0.11–0.35	4.10–4.95/0.05–0.18
	Operability (*M/CV*)	2.80–4.65/0.11–0.25	4.00–5.00/0.00–0.18
Evaluation consistency	Kendall’s *W*	0.211 (χ^2^ = 198.78, *P* < 0.001)	0.251 (χ^2^ = 235.48, *P* < 0.001)

*M*, mean; *CV*, coefficient of variation.

**FIGURE 1:**
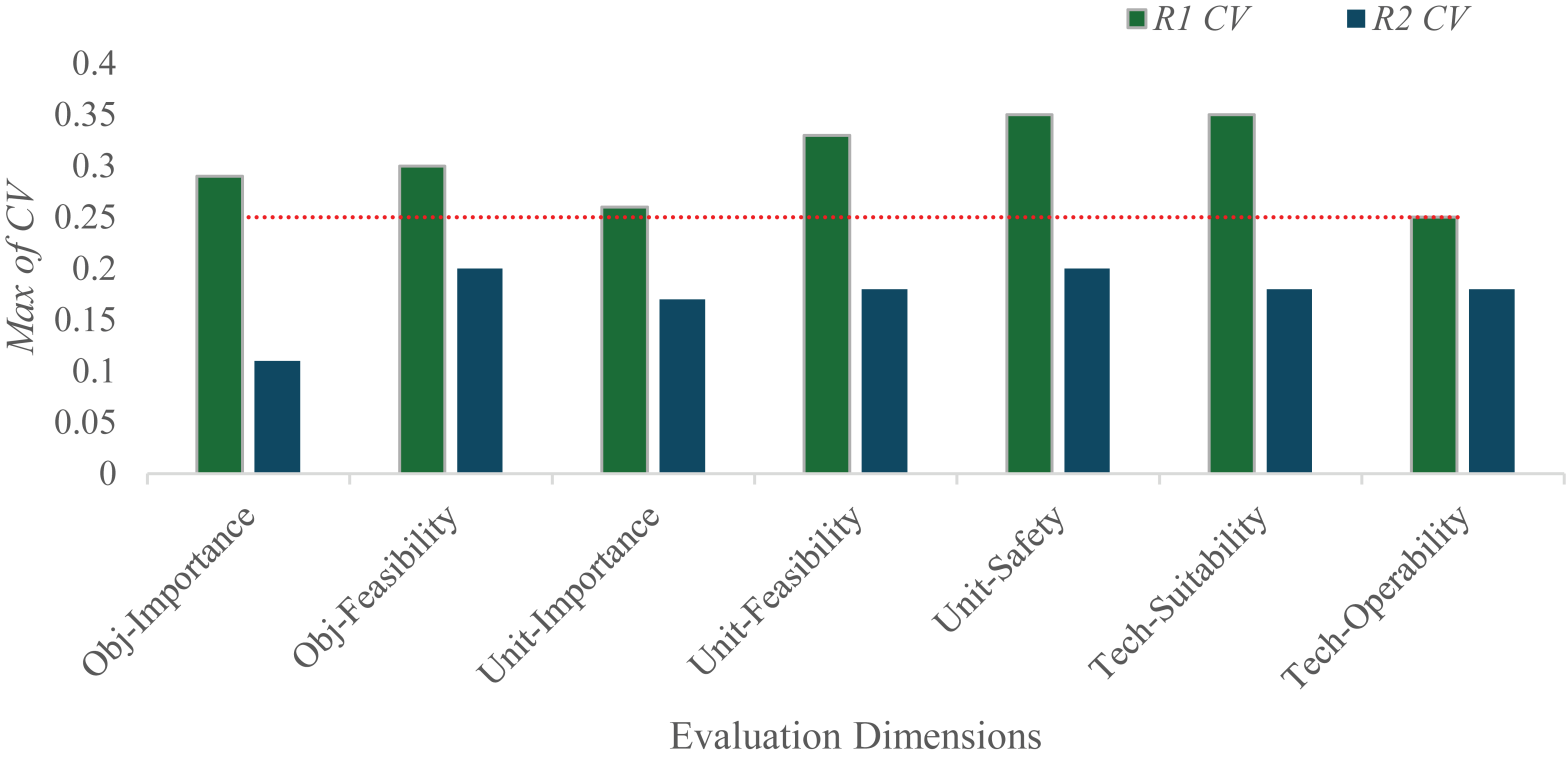
Comparison of consensus levels between Round 1 and Round 2 of the Delphi consultation.

As shown in Figure 1, the bar chart illustrates the maximum CV across evaluation dimensions for both consultation rounds. The red dashed line indicates the predefined consensus threshold (CV = 0.25). Compared with Round 1, the maximum CV values in Round 2 exhibited a marked decrease, with all indicators in the final round falling securely below the threshold. This trend indicates that the expert opinions achieved a satisfactory level of convergence and consistency regarding the program’s structure and content.

### Refinement of program indicators

Qualitative feedback from 10 experts in Round 1 was analyzed to refine the initial program draft. Three guiding principles emerged to inform the optimization process. Safety Principle: Given the heightened rejection sensitivity characteristics of adolescents with anxiety and depression, high-competition or fast-paced activities were removed. Cognitive Adaptation Principle: In consideration of the reduced cognitive processing speed associated with depressed states, the complexity of SFBT questioning was adjusted to emphasize the achievement of micro-successes. Ethics and Boundaries Principle: To maintain clinical boundaries, the therapeutic focus was redirected from fostering personal social connections within the group toward facilitating the transfer of interpersonal skills to real-world contexts.

Revisions based on these principles are summarized in Table 3.

**TABLE 3:** Qualitative expert feedback and evidence-based program refinements.

Dimension	Initial draft	Expert suggestions	Revised program	Refinement principles
Goal setting	Establishing long-term connections within the group.	Due to ethical and boundary requirements, private social relationships outside the group are generally discouraged.	Facilitating the transfer of group experiences to real-world interpersonal relationships.	Ethical boundaries: attachment theory
Session activities	Session 5: musical chairs (warm-up).	Highly competitive and fast-paced activities may trigger social stress or acute anxiety in sensitive adolescents.	Revised to: gentle balloon release (a collaborative task).	Safety principle: social evaluative threat theory
	Session 6: overloaded schedule (e.g., “Emotion Express”).	Adolescents with reduced cognitive processing speeds may struggle to complete multiple deep discussions within time limits.	Removed emotion express to allocate sufficient time for core SFBT dialogues.	Cognitive adaptation: cognitive load theory
	Session 7: “Balloon Battle” (warm-up).	Suddenly loud noises pose a potential risk for members prone to panic attacks.	Revised to: balloon floating (shifting from competition to cooperation).	Safety principle: trauma-informed care (TIC)
	Session 8: “Crossing the River” (main activity).	Excessive or forced physical contact may trigger defensive reactions in adolescents with past trauma.	Revised to: red string web (using visual connection instead of physical proximity).	Safety principle: psychological boundary theory
Technical application	Standard miracle question.	May appear overly optimistic or unrealistic to those with severe helplessness, potentially inducing resistance.	Adapted to the small change hypothesis (e.g., imagining a 0.5-point improvement).	Cognitive adaptation: perceived control theory

### Finalization of the intervention program

The final validated program comprises eight functional units and seven core SFGT techniques. The structure focuses on transitioning from interpersonal competition to social support, aiming to enhance interpersonal efficacy while maintaining clinical safety (Table 4).

**TABLE 4:** Solution-Focused Group Therapy (SFGT) program for adolescents with anxiety and depression.

Unit	Theme	Core objectives	Key activities	SFGT techniques
1	Nice to meet you	To alleviate initial social anxiety and foster a preliminary sense of group safety and trust.	Warm-up: big wind blows	Goal setting, normalization
			Main: Name Tag Creation, Snowballing	
			Closing: Group Contract Signing, Goal Sharing	
2	Cooperation and mutual growth	To enhance collaborative awareness and strengthen confidence in managing interpersonal challenges.	Warm-up: squirrels and trees, whack-a-mole	Scaling questions, exception seeking, compliments
			Main: homework review, bag of tricks	
			Closing: intricate knots	
3	Discovering the inner self	To encourage self-acceptance and assist members in identifying personal interpersonal strengths.	Warm-up: who is this	Concretization, tracking questions, compliments
			Main: the unique orange, talent showcase	
			Closing: positive feedback session	
4	Connecting through communication	To experience the dynamics of two-way communication and practice non-violent expression.	Warm-up: flower and leaves	Snowballing, scaling questions, first sign
			Main: dictation drawing, navigating the minefield	
			Closing: mirroring reflection	
5	Conflict and collaboration	To practice teamwork in a non-competitive environment and learn to express needs and boundaries.	Warm-up: gentle balloon release	Compliments, scaling questions
			Main: defining boundaries, rapid 60-second challenge, spaghetti tower	
			Closing: conflict case review	
6	Dancing with emotions	To improve acceptance of negative emotions and understand the underlying psychological needs.	Warm-up: number-name call out	Exception seeking, miracle/hypothetical questions, normalization
			Main: inside ou, tree in the rain	
			Closing: sharing emotion regulation strategies	
7	My social world	To identify and activate existing peer and family support networks in daily life.	Warm-up: floating balloons	Compliments, hypothetical questions
			Main: homework review, social network mapping, OH cards with WOOP strategy	
			Closing: Envisioning Future Growth	
8	Managing separation	To reinforce successful experiences gained in the group and facilitate the transition of safety into real-world social motivation.	Warm-up: rhythmic clapping exercise	Normalization, EARS (elicit, amplify, reinforce, start again)
			Main: final homework review, sincere 100 s, red string web	
			Closing: blessing cards	

## Discussion

The current study developed a structured SFGT program targeting interpersonal impairment through a rigorous consensus-building process. The findings indicate that both the authority coefficient (*Cr* = 0.90) and the degree of consensus among experts (*W* = 0.251, *p* < 0.001) meet the established benchmarks for content validity. Although Kendall’s *W* increased from a low level (0.211) to a moderate level (0.251), this change-while statistically significant-did not reach the strong consensus threshold (*W* > 0.5). This moderate level of agreement is consistent with the inherent complexity of designing a multifaceted group therapy protocol and reflects the diverse clinical perspectives within the multidisciplinary panel.

Adolescents with anxiety and depression frequently experience the “island effect”, a phenomenon characterized by profound social isolation and internalized stigma (Bieleninik et al., 2017). By integrating SFBT’s normalization techniques within the first two units, the program reframes emotional distress as a common response to situational stressors rather than a personal deficit. This reframing facilitates the activation of universality—a core therapeutic factor in group therapy—which may help mitigate the severe self-criticism often reinforced by high-control Chinese family environments (Kraines and Wells, 2017). Furthermore, the role transition from a help-seeker to helper in Unit 3, empowers participants to rediscover their self-identity through altruistic engagement within the group.

The expert panel’s skepticism regarding competitive activities in the initial draft highlights a critical clinical reality: the heightened rejection sensitivity (RS) characteristic of adolescents with internalizing disorders. Traditional competitive tasks can inadvertently trigger acute stress responses or fight-or-flight reactions in vulnerable individuals, potentially leading to therapeutic re-traumatization (Porges, 2025). Accordingly, these activities were replaced with collaborative tasks such as the Gentle Balloon Release with the aim of fostering a corrective emotional experience. By navigating interpersonal challenges within a non-threatening environment, group members would ideally receive collaborative feedback that contrasts with their previous traumatic interpersonal scripts (Nakamura and Iwakabe, 2018). This strategic shift from confrontation to cooperation is hypothesized to provides the psychological scaffolding necessary for adolescents to relearn trust. Similarly, replacing forced physical proximity with visual connection respects the psychological boundaries of members, thereby preventing defensive withdrawal during the early stages of group formation (Ringwald et al., 2025).

A pivotal refinement in this protocol is the adaptation of the Miracle Question into the Small Change Hypothesis. Experts observed that for adolescents experiencing psychomotor retardation or reduced mental energy, the cognitive demands required to envision a “miraculous” future often prohibitive (van Tilburg et al., 2025). This modification aligns with Perceived Control Theory, which posits that narrowing the focus to incremental, manageable shifts reduces the risk of frustration (Zhang et al., 2022). By lowering the threshold for success, the program is intended to enable participants to identify exceptions even in the context of severe depressive states, which would theoretically enhance the intervention’s clinical viability in real-world scenarios where psychological energy is limited.

### Contextual applicability of the protocol

Regarding the contextual applicability of this protocol, while the expert panel was primarily recruited from hospital-based settings, the final protocol embodies a de-pathologized, postmodern orientation that is inherently suited for a wide range of service settings. The emphasis on strengths rather than deficits, the collaborative rather than prescriptive stance, and the focus on transferable interpersonal skills rather than symptomatic cure make this protocol equally applicable in school counseling centers, community mental health services, and hospital outpatient departments. Indeed, the adaptation of core techniques—such as replacing the Miracle Question with the Small Change Hypothesis—was specifically designed to reduce the stigma associated with mental health interventions, making the program more accessible to adolescents and families who may be reluctant to seek help in formal psychiatric settings. Future implementation studies will systematically evaluate the protocol’s feasibility and acceptability across these diverse contexts.

### Dose rationale

While SFBT has been implemented with varying session lengths—including 2–4 sessions for online adolescent anxiety protocols (Chen et al., 2023), 4-week programs for young adult cancer patients (Zhang et al., 2022), and six-session formats in community youth mental health services—the eight-session structure adopted here was derived from two sources: (1) the eight interpersonal themes identified in our initial literature synthesis and confirmed by expert consensus, and (2) practical considerations regarding typical outpatient treatment durations in Chinese clinical settings. Nonetheless, the optimal dose remains an empirical question. Future research should systematically investigate dose-response relationships through factorial or stepped-care trial designs.

### Limitations and future directions

While the Delphi consensus provides a robust foundation for content validity, several limitations should be acknowledged.

First, the geographic concentration of the expert panel within Henan Province reflects an intentional focus on this specific high-pressure educational environment. While this provides unique insights into a highly representative demographic of Chinese adolescents under extreme academic pressure, the program’s generalizability to coastal regions, rural areas, or ethnic minority communities requires further investigation. Future multi-center validation studies across provinces are needed to assess the protocol’s adaptability to diverse Chinese subcultures and educational systems.

Second, the current findings represent content validation only. The hypothesized clinical benefits remain theoretical mechanisms derived from expert consensus and existing literature. Empirical evidence from pilot feasibility studies and fully powered randomized controlled trials (RCTs) is essential to establish clinical efficacy. Ongoing research includes an RCT examining the effects of this SFGT protocol on peer friendship quality in adolescents with anxiety disorders (Zhuang et al., 2026) and two additional trials currently under review. The dose–response relationship and long-term efficacy of the protocol also require rigorous quantification through future multi-center RCTs (Berger et al., 2013; Moher et al., 2012).

Third, the expert panel did not include adolescents, parents, or school counselors—a deliberate design choice consistent with the Delphi method’s focus on clinical and academic expertise for initial content validation. However, the absence of these stakeholder perspectives constitutes a significant limitation. Adolescents’ subjective experiences and parents’/school counselors’ implementation insights cannot be fully captured through expert judgment alone. To address this, our research team is actively collecting feedback from adolescent participants during the ongoing randomized controlled trial of this SFGT protocol. This iterative process of gathering user input will inform ongoing refinements to the intervention, ensuring that the protocol evolves in response to real-world acceptability and feasibility data prior to broader dissemination.

Fourth, the Delphi technique is designed for expert consensus-building, not for end-user acceptability testing. While this approach was appropriate for the formative stage of protocol development, future research should assess the program’s acceptability and feasibility directly with target users (adolescents and families) across both clinical and school settings. Additionally, the longitudinal stability of interpersonal gains and the program’s scalability within broader Chinese school and clinical settings remain to be examined.

## Conclusion

This study successfully developed and content-validated a culturally sensitive, eight-session Solution-Focused Group Therapy program for Chinese adolescents with anxiety and depression, using a two-round Delphi expert consensus process. The final protocol embodies three innovations: (1) systematic exclusion of competitive activities to accommodate heightened rejection sensitivity; (2) adaptation of the Miracle Question into the Small Change Hypothesis for adolescents with diminished cognitive processing speed; and (3) a structured framework bridging group therapeutic factors with real-world interpersonal skill transfer. The program demonstrates preliminary content validity and theoretical plausibility as a de-pathologized, strengths-based intervention suitable for clinical and community settings. Future research will focus on empirical validation through pilot feasibility studies, user acceptability testing with adolescents and parents, and multi-center randomized controlled trials to establish clinical efficacy and optimal implementation strategies.

## Data Availability

The raw data supporting the conclusions of this article will be made available by the authors, without undue reservation.
