# Supplementary material for: Development and validation of a Solution-Focused Group Therapy program for interpersonal relationships in adolescents with anxiety and depression: a Delphi-based consensus study
Source: Front Psychol. 2026 Jul 22;17:1834496. doi: 10.3389/fpsyg.2026.1834496 (PMC13437402; doi:10.3389/fpsyg.2026.1834496)
Supplement: Supplementary file 1 [file Supplementary_file_1.docx]

# **焦虑抑郁青少年人际关系焦点解决团体治疗方案专家咨询表（一）**

尊敬的专家：

您好！我们诚挚地邀请您参与构建科学、可操作的《焦虑抑郁青少年人际关系焦点解决团体治疗方案》，为临床干预焦虑抑郁青少年的人际关系障碍（如社交回避、沟通误解、冲突处理困难等）提供标准化框架。方案基于焦点解决短期治疗（SFBT）理论、团体动力学原理，结合焦虑抑郁青少年心理特点，初步设计“8单元结构化团体”框架（每次1.5-2小时，每周1次，共8次）。

现通过第一轮德尔菲专家咨询，邀请您从专业视角评估方案的合理性、实操性与青少年适配性。若您深耕青少年心理治疗、焦点解决疗法、团体干预任一领域，可聚焦熟悉板块反馈；若多领域均有研究，欢迎全面建言。本研究将依据专家评分的均数、变异系数及意见趋同性进行指标筛选。您的意见将直接影响方案的优化方向，感谢您的支持！

课题负责人：张丽霞

联系人：谷亚男18406565339；

王雪云16638068391

**表一、专家基本情况表**

1.性别：①男；②女

2.年龄：

3.最高学历：①大专及以下；②本科；③硕士；④博士及以上

4.专业技术职称：①初级（如初级心理治疗师、住院医师）；②中级（如中级心理治疗师、主治医师）；③副高（如副主任心理师/医师）；④正高（如主任心理师/医师）；⑤其他（请注明：__________）

5.您的专业：①精神病学；②心理学；③教育学；④其他

6.您从事该专业工作年限：

7.工作单位类型：①高校（心理学/医学专业）；②精神专科医院（心理科/儿少科）；③综合医院（心理科/儿科）；④青少年心理健康服务机构；⑤科研院所；⑥其他（请注明：__________）

8.您是否接受过焦点解决短期治疗（SFBT）的相关培训或认证？①是；②否。

**表二、方案核心目标咨询表**

本方案针对的青少年群体为：符合 DSM-5或 ICD-10诊断标准，处于焦虑或抑郁状态，且在HAMA/HAMD量表测评中达中度及以上水平，同时伴有人际关系困扰者。

1.方案总目标：提升焦虑抑郁青少年的友谊质量，降低孤独感；分目标涵盖“关系建立、技能提升、自信培养、情绪适配”四大维度。

2.评分标准：重要性（1=很不重要，2=较不重要，3=一般，4=较重要，5=非常重要）；可行性（1=很不可行，2=较不可行，3=一般，4=较可行，5=非常可行）；

3.请在“修改建议”栏说明理由；补充目标需同步评分。

| 序号 | 初步拟定方案目标 | 重要性（1-5分） | 可行性（1-5分） | 修改/补充建议 |
| --- | --- | --- | --- | --- |
| 1 | 营造安全团体氛围：帮助青少年建立信任，明确团体规则（如隐私保密、尊重表达） |  |  |  |
| 2 | 提升人际认知：引导青少年识别自身人际优势（如倾听、共情）与核心困扰（如回避、冲突） |  |  |  |
| 3 | 掌握沟通技能：通过实操训练，让青少年学会 “清晰表达需求”“有效倾听回应”“非暴力沟通” |  |  |  |
| 4 | 增强冲突解决能力：培养 “换位思考”“协商妥协”“危机冷静处理” 的策略与意识 |  |  |  |
| 5 | 构建支持网络：帮助青少年激活现有支持资源（如同伴、家人、老师），建立长期人际联结 |  |  |  |
| 6 | 补充目标：__________________________ |  |  |  |

**表三、方案核心单元与活动专家评估表**

填写说明：方案共8个单元（如下表），每个单元含“热身活动、主要活动、结束活动”，配套焦点解决技术与家庭作业。请评估单元逻辑、活动适配性（如青少年兴趣、认知水平）、时长合理性，评分标准同“目标评估表”。

| 单元序号 | 单元主题 | 初步拟定方案目标 | 重要性（1-5 分） | 可行性（1-5 分） | 安全性（1-5 分） | 修改建议 |
| --- | --- | --- | --- | --- | --- | --- |
| 1 | Nice to meet you | 1. 热身：大风吹（成员围圈互动，缓解陌生感） |  |  |  |  |
|  |  | 2. 主要：写名牌（自制昵称名牌 + 自我介绍）、滚雪球（分组复述成员信息） |  |  |  |  |
|  |  | 3. 结束：签署团体契约 + 分享参与目标 |  |  |  |  |
|  |  | **焦点解决技术**：目标架构、一般化 |  |  |  |  |
| 2 | 互帮互助友谊进步 | 1. 热身：松鼠大树（三人组队互动，增强协作）、棒打薄情郎（纸棒游戏强化成员记忆） |  |  |  |  |
|  |  | 2. 主要：回顾家庭作业（分享人际困扰场景）、锦囊妙计（分组提解决方案） |  |  |  |  |
|  |  | 3. 结束：心有千千结（拉手解圈，强化信任） |  |  |  |  |
|  |  | **焦点解决技术**：评量询问、例外询问、赞美 |  |  |  |  |
| 3 | 看见发光的自己 | 1. 热身：猜猜他是谁（头戴姓名卡，提问猜人） |  |  |  |  |
|  |  | 2. 主要：我这个橘子（观察橘子特征，类比自我接纳）、天生我才（填写优势表格 + 小组分享） |  |  |  |  |
|  |  | 3. 结束：戴高帽（成员互夸，增强自信） |  |  |  |  |
|  |  | **焦点解决技术**：具体化、追踪询问、赞美 |  |  |  |  |
| 4 | 沟通你我他 | 1. 热身：鲜花绿叶（分组互动，强化倾听） |  |  |  |  |
|  |  | 2. 主要：你说我画（单向 / 双向沟通对比）、巧过地雷阵（蒙眼引导，培养信任） |  |  |  |  |
|  |  | 3. 结束：镜面反射（动作模仿，提升共情） |  |  |  |  |
|  |  | **焦点解决技术**：滚雪球、评量询问、First Sign |  |  |  |  |
| 5 | 冲突与合作 | 1. 热身：抢椅子（分组竞赛，活跃氛围） |  |  |  |  |
|  |  | 2. 主要：圈地划界（空间守护，学习表达需求）、急速 60 秒（数字卡片排序，培养协作）、面条筑塔（团队筑塔，提升创造力） |  |  |  |  |
|  |  | 3. 结束：冲突案例复盘 |  |  |  |  |
|  |  | **焦点解决技术**：赞美、评量询问 |  |  |  |  |
| 6 | 与情绪共舞 | 1. 热身：抢数字报名字（紧张互动，引入情绪主题） |  |  |  |  |
|  |  | 2. 主要：情绪传达室（表情传递，识别情绪）、头脑特工队（观看片段，理解情绪需求）、雨中树（绘画分析，探索压力） |  |  |  |  |
|  |  | 3. 结束：情绪调节技巧分享 |  |  |  |  |
|  |  | **焦点解决技术**：例外询问、假设询问、一般化 |  |  |  |  |
| 7 | 我的小世界 | 1. 热身：气球大战（组队踩气球，增强联结） |  |  |  |  |
|  |  | 2. 主要：回顾家庭作业（分享人际改变）、人际你我他（梳理人际网络）、OH 卡 WOOP（目标可视化） |  |  |  |  |
|  |  | 3. 结束：未来期待分享 |  |  |  |  |
|  |  | **焦点解决技术**：赞美、假设询问 |  |  |  |  |
| 8 | 处理分离 | 1. 热身：拍拍操（按摩互动，强化情感联结） |  |  |  |  |
|  |  | 2. 主要：回顾家庭作业（分享感恩表达）、真情 100 秒（对视沟通，深化信任）、同舟共济（报纸站立，强化协作） |  |  |  |  |
|  |  | 3. 结束：祝福心意卡（互赠卡片，处理离别） |  |  |  |  |
|  |  | **焦点解决技术**：一般化、EARS |  |  |  |  |

表四、焦点解决技术适配性评估表

方案核心技术已融入各单元，请评估技术与青少年的适配性（如是否易懂、是否能引导改变）、可操作性（如带领者是否易掌握），评分标准：适配性（1 = 很不适配，2 = 较不适配，3 = 一般，4 = 较适配，5 = 非常适配）；可操作性（1 = 很不可操作，2 = 较不可操作，3 = 一般，4 = 较可操作，5 = 非常可操作）。

| **序号** | **焦点解决技术** | **应用单元 / 场景示例** | **适配性**  **（1-5 分）** | **可操作性（1-5 分）** | **修改建议（如技术简化、话术调整）** |
| --- | --- | --- | --- | --- | --- |
| 1 | 目标架构技术 | 单元 1（询问参与目标：“你希望通过团体获得什么？”） |  |  |  |
| 2 | 例外询问技术 | 单元 2（探索成功经验：“你之前人际顺利时做了什么？”） |  |  |  |
| 3 | 评量询问技术 | 单元 4（沟通效果评分：“你认为本轮沟通相似度是几分？”） |  |  |  |
| 4 | 赞美技术 | 单元 3（成员互夸：“你主动分享的勇气很珍贵”） |  |  |  |
| 5 | 假设询问技术 | 单元 6（奇迹提问：“若人际问题解决，你会有什么不同？”） |  |  |  |
| 6 | First Sign 技术 | 单元 4（小步改变：“你能做的第一个沟通改变是什么？”） |  |  |  |
| 7 | EARS 技术 | 单元 8（经验强化：通过追问引出改变迹象、放大积极效应、强化成功经验，并引导循环尝试） |  |  |  |

表五、专家权威程度调查

5.1 主题熟悉度评估

| 研究主题 | 很熟悉 | 熟悉 | 较熟悉 | 一般 | 不熟悉 |
| --- | --- | --- | --- | --- | --- |
| 焦虑抑郁青少年人际关系干预 |  |  |  |  |  |
| 焦点解决团体治疗在青少年中的应用 |  |  |  |  |  |

5.2判断依据评估

| 判断依据 | 专家自我评价判断依据程度 | | |
| --- | --- | --- | --- |
|  | 大 | 中 | 小 |
| 理论分析 |  |  |  |
| 实践经验 |  |  |  |
| 参考国内外有关资料 |  |  |  |
| 直觉 |  |  |  |

本次问卷完全匿名，数据仅用于学术研究，符合伦理规范，感谢您的大力支持与帮助！

联系人:谷亚男18406565339；王雪云16638068391

后续将汇总专家意见形成第二轮咨询表，届时会同步至您本人，敬请关注。

再次感谢您的专业贡献！
