# Supplementary material for: Development and validation of a Solution-Focused Group Therapy program for interpersonal relationships in adolescents with anxiety and depression: a Delphi-based consensus study
Source: Front Psychol. 2026 Jul 22;17:1834496. doi: 10.3389/fpsyg.2026.1834496 (PMC13437402; doi:10.3389/fpsyg.2026.1834496)
Supplement: Supplementary file 2 [file Supplementary_file_2.docx]

# **焦虑抑郁青少年人际关系焦点解决团体治疗方案专家咨询表（二）**

尊敬的专家：

您好！在您的大力支持与悉心指导下，本课题组已顺利完成《焦虑抑郁青少年人际关系焦点解决团体治疗方案》的第一轮专家咨询工作。在第一轮咨询中，各位专家针对方案的安全性、认知负荷及临床伦理提出了极具建设性的修改建议。课题组经过深度讨论与理论复核，对方案进行了如下关键优化，请您在评价第二轮表单时重点审阅：

针对抑郁青少年可能的社交退缩与生理防御，我们取消了具有竞争压力的活动（抢椅子）和过度身体接触的环节（同舟共济），代之以气球轻放与红线结网。旨在通过非侵入式的协作，构建安全的团体容器。针对青少年在情绪低落期可能出现的注意力和思维加工减慢，我们对第6节情绪单元进行改善。删除了耗时较长的观影与多重手工环节，预留更多空间进行SFBT深度对话，确保干预的质量。将传统的奇迹提问改良为微小改变的假设。这一调整旨在降低改变的心理门槛，引导成员识别生活中已存在的微小成功，以应对严重的习得性无助。将原有的建立长期人际联结目标修正为经验向现实关系的迁移。重点在于辅助成员利用团体中习得的沟通技巧，激活其原有的家庭与同伴支持网络。

第二轮专家咨询需要请您再次对方案的核心目标、各单元活动及技术的适配性进行多维度评价。您的意见对我们的研究工作非常重要。在调查期间，如您对本研究感兴趣，或有任何疑问，请随时与我们联系。在此，再次诚挚地感谢您对本研究的大力支持和帮助。

祝您和您的家人身体健康，万事如意!

课题负责人： 张丽霞

联系人： 谷亚男 18406565339；王雪云 16638068391

**表一、方案核心目标咨询表**

1.目标修改标准

根据专家评价及建议进行修改。保留标准为目标重要性和可行性＞4且变异系数＜0.25的目标。

2.目标修改情况

构建支持网络：专家认为基于伦理和边界的要求，通常不鼓励成员在团体之外建立私人社交关系（以防形成小团体或二次伤害）。虽然目标是提升现实生活的人际能力，但“在团体中建立长期联结”在临床伦理上存在争议。建议修改为“将团体中获得的经验迁移至现实人际关系中”。

3.评分标准：重要性（1=很不重要，2=较不重要，3=一般，4=较重要，5=非常重要）；可行性（1=很不可行，2=较不可行，3=一般，4=较可行，5=非常可行）；

4.请在“修改建议”栏说明理由；补充目标需同步评分。

| 序号 | 初步拟定方案目标 | 重要性（1-5分） | | 可行性（1-5分） | | 修改/补充建议 |
| --- | --- | --- | --- | --- | --- | --- |
|  |  | 上一轮评分均分 | 本轮评分 | 上一轮评分均分 | 本轮评分 |  |
| 1 | 营造安全团体氛围：帮助青少年建立信任，明确团体规则（如隐私保密、尊重表达） | 4.75 |  | 4.65 |  |  |
| 2 | 提升人际认知：引导青少年识别自身人际优势（如倾听、共情）与核心困扰（如回避、冲突） | 4.3 |  | 4.8 |  |  |
| 3 | 掌握沟通技能：通过实操训练，让青少年学会 “清晰表达需求”“有效倾听回应”“非暴力沟通” | 4.7 |  | 4.3 |  |  |
| 4 | 增强冲突解决能力：培养 “换位思考”“协商妥协”“危机冷静处理” 的策略与意识 | 4.4 |  | 4.4 |  |  |
| 5 | 构建支持网络：帮助青少年激活现有支持资源（如同伴、家人、老师），将团体中获得的经验迁移至现实人际关系中。 | 3.9 |  | 4.2 |  | 已根据第一轮意见重构目标描述 |
| 6 | 补充目标：__________________________ |  |  |  |  |  |

**表三、方案核心单元与活动专家评估表**

1.方案修改标准

根据专家评价及建议进行修改。

2.方案修改情况

第五期冲突与合作：“抢椅子”改为“气球轻放”。实施过程为小组成员围圈，任务是让气球尽可能久地保持在空中不落地。专家认为焦虑抑郁青少年通常伴有社交焦虑、躯体化症状或对他人评价的高度敏感。抢椅子这种高竞争性、快节奏的活动可能导致原本就敏感的青少年产生强烈的挫败感或社交压力，甚至引发焦虑急性发作。建议将人与人的竞争转变为团队共同解决任务，降低焦虑青少年的社交压力。

第六期与情绪共舞，删除“情绪传达室”。专家认为1.5-2小时内完成这么多环节，往往只能走马观花，无法深入讨论 SFBT 的核心。而且抑郁状态下的青少年往往存在注意力不集中、认知加工速度减慢的情况。这一单元涵盖了观影、绘画和三种SFBT 技术，内容极其饱和。删除了活动适量，保留了核心体验活动，保证SFBT深度对话的时间。

第七期我的小世界，“气球大战”改为“气球漂浮”。实施过程为带领者向圆圈中心抛出一个气球，要求成员合力让气球保持在空中不落地，当成员逐渐熟悉节奏后，带领者逐个加入第2个气球。要求成员在全程不说话的情况下，继续保持所有气球不落地。专家认为踩气球的爆破声对焦虑、惊恐倾向的青少年有潜在诱发风险。

第八期处理分离：“同舟共济”改为“红线结网”。 实施过程为成员围坐，手持红线团。第一人分享在团体中的收获后，抓住线头将球抛给下一人，最终形成一张密集的红线网。结合专家想法，专家认为同舟共济需要成员在极小的空间内身体紧贴。对于受过心理创伤或有抑郁心境的青少年，过近的身体接触可能引起生理不适或防御反应。用视觉上的联结（线）代替物理上的挤压，既保留了深度的情感联结，又尊重了青少年的身体边界。

3. 填写说明：方案共8个单元（如下表），每个单元含“热身活动、主要活动、结束活动”，配套焦点解决技术与家庭作业。请评估单元逻辑、活动适配性（如青少年兴趣、认知水平）、时长合理性和安全性，评分标准同“目标评估表

| 单元序号 | 单元主题 | 初步拟定方案目标 | 重要性（1-5 分） | | 可行性（1-5 分） | | 安全性（1-5分） | | 修改建议 |
| --- | --- | --- | --- | --- | --- | --- | --- | --- | --- |
|  |  |  | 上一轮均分 | 本轮评分 | 上一轮均分 | 本轮评分 | 上一轮均分 | 本轮评分 |  |
| 1 | Nice to meet you | 1.热身：大风吹（成员围圈互动，缓解陌生感）  2.主要：写名牌（自制昵称名牌+自我介绍）、滚雪球（分组复述成员信息）  3.结束：签署团体契约+分享参与目标  焦点解决技术：目标架构、一般化 | 4.8 |  | 4.55 |  | 4.55 |  |  |
| 2 | 互帮互助友谊进步 | 1.热身：松鼠大树（三人组队互动，增强协作）、棒打薄情郎（纸棒游戏强化成员记忆）  2.主要：回顾家庭作业（分享人际困扰场景）、锦囊妙计（分组提解决方案）  3.结束：心有千千结（拉手解圈，强化信任）  焦点解决技术：评量询问、例外询问、赞美 | 4.65 |  | 4.6 |  | 4.6 |  |  |
| 3 | 看见发光的自己 | 1.热身：猜猜他是谁（头戴姓名卡，提问猜人）  2. 主要：我这个橘子（观察橘子特征，类比自我接纳）、天生我才（填写优势表格 + 小组分享）  3.结束：戴高帽（成员互夸，增强自信）  焦点解决技术：具体化、追踪询问、赞美 | 4.55 |  | 4.55 |  | 4.5 |  |  |
| 4 | 沟通你我他 | 1.热身：鲜花绿叶（分组互动，强化倾听）  2.主要：你说我画（单向 / 双向沟通对比）、巧过地雷阵（蒙眼引导，培养信任）  3.结束：镜面反射（动作模仿，提升共情）  焦点解决技术：滚雪球、评量询问、First Sign | 4.6 |  | 4.35 |  | 4.65 |  |  |
| 5 | 冲突与合作 | 1.热身：气球轻放（活跃氛围）  2.主要：圈地划界（空间守护，学习表达需求）、急速 60 秒（数字卡片排序，培养协作）、面条筑塔（团队筑塔，提升创造力）  3.结束：冲突案例复盘  焦点解决技术：赞美、评量询问 | 4.35 |  | 4.05 |  | 3.5 |  |  |
| 6 | 与情绪共舞 | 1.热身：抢数字报名字（紧张互动，引入情绪主题）  2.主要：头脑特工队（观看片段，理解情绪需求）、雨中树（绘画分析，探索压力）  3.结束：情绪调节技巧分享  焦点解决技术：例外询问、假设询问、一般化 | 4.55 |  | 3.55 |  | 4.45 |  |  |
| 7 | 我的小世界 | 1.热身：气球漂浮（组队增强联结）  2.主要：回顾家庭作业（分享人际改变）、人际你我他（梳理人际网络）、OH卡WOOP（目标可视化）  3.结束：未来期待分享  焦点解决技术：赞美、假设询问 | 4.45 |  | 3.25 |  | 3.5 |  |  |
| 8 | 处理分离 | 1.热身：拍拍操（按摩互动，强化情感联结）  2.主要：回顾家庭作业（分享感恩表达）、真情 100秒（对视沟通，深化信任）、红线结网（强化协作）  3.结束：祝福心意卡（互赠卡片，处理离别）  焦点解决技术：一般化、EARS | 4.05 |  | 4.7 |  | 3.75 |  |  |

表四、焦点解决技术适配性评估表

1.方案修改标准

根据专家评价及建议进行修改。

2.方案修改情况

假设询问技术：专家认为假设询问对处于习得性无助严重的青少年来说，有时显得过于乐观和不切实际。如果心理治疗师功底不足，青少年可能会感到你根本不理解我的痛苦，从而导致阻抗。建议改为“微小改变的假设”，如“想象明天醒来，即便焦虑还在，但如果那份沉重感减轻了 0.5 分，你会观察到自己有什么微小的不同？”

3.请评估技术与青少年的适配性（如是否易懂、是否能引导改变）、可操作性（如带领者是否易掌握），评分标准：适配性（1 = 很不适配，2 = 较不适配，3 = 一般，4 = 较适配，5 = 非常适配）；可操作性（1 = 很不可操作，2 = 较不可操作，3 = 一般，4 = 较可操作，5 = 非常可操作）。

| **序号** | **焦点解决技术** | **应用单元 / 场景示例** | **适配性**  **（1-5 分）** | **可操作性（1-5 分）** | **修改建议（如技术简化、话术调整）** |
| --- | --- | --- | --- | --- | --- |
| 1 | 目标架构技术 | 单元 1（询问参与目标：“你希望通过团体获得什么？”） |  |  |  |
| 2 | 例外询问技术 | 单元 2（探索成功经验：“你之前人际顺利时做了什么？”） |  |  |  |
| 3 | 评量询问技术 | 单元 4（沟通效果评分：“你认为本轮沟通相似度是几分？”） |  |  |  |
| 4 | 赞美技术 | 单元 3（成员互夸：“你主动分享的勇气很珍贵”） |  |  |  |
| 5 | 假设询问 | 单元 6（微小改变的假设：“想象明天醒来，即便焦虑还在，但如果那份沉重感减轻了 0.5 分，你会观察到自己有什么微小的不同？”） |  |  |  |
| 6 | First Sign 技术 | 单元 4（小步改变：“你能做的第一个沟通改变是什么？”） |  |  |  |
| 7 | EARS 技术 | 单元 8（经验强化：通过追问引出改变迹象、放大积极效应、强化成功经验，并引导循环尝试） |  |  |  |

本次问卷到此结束，感谢您的大力支持与帮助！

联系人:谷亚男18406565339；王雪云16638068391
